# Estimating the Global Burden of Snakebite Can Help To Improve Management

**DOI:** 10.1371/journal.pmed.0050221

**Published:** 2008-11-04

**Authors:** Jean-Philippe Chippaux

## Abstract

Jean-Philippe Chippaux discusses the implications and limitations of a new study on the global burden of snakebite.

## Lack of Antivenom: A Market Failure

Snakebite is a common medical emergency in developing countries in tropical regions. The only specific treatment is antivenom [1], but this is often unavailable in remote health centres, due to market failure [2,3].

In the 1980s in Africa, 150,000 to 200,000 doses of antivenom were sold annually, whereas current sales have fallen to less than 20,000 doses per year. The price of a vial of antivenom has risen by a multiple of 10 over the last 20 years. Today, with the global economic crisis, the treatment represents several months of the average income of rural families. In an emergency situation, there is insufficient time for families to sell their crops and livestock to buy the antivenom.

The main reasons that the antivenom manufacturers use to justify such a dramatic price increase are the complexity and cost of antivenom production. But there are three other reasons for the escalating costs. First, the antivenom market is unstable. Second, there is little financial incentive for pharmacists and health centres to sell antivenom because they reap only feeble profit margins. Finally, there are no comprehensive data on how many doses of antivenom are required and where they should be distributed.

## Estimating the Global Burden

The first assessment of global snakebite incidence and mortality was undertaken by Swaroop and Grab in 1954 [4]. They estimated the number of snakebites (in fact envenomings at that time) and deaths respectively at 500,000 and 30,000–40,000 per year (their estimates excluded China, USSR, and central European countries, from which data were unavailable). These figures certainly underestimated the true burden because Swaroop and Grab lacked relevant information for their assessment. The second assessment, which I published in 1998, was based on a greater number of publications and so was more reliable, but many information gaps remained, including the question of how representative the local studies were of the wider epidemiological situation [5]. Despite these gaps, it has generally been assumed that there are about 5 million snakebites worldwide each year, leading to 125,000 deaths.

**Linked Research Article**

This Perspective discusses the following new study published in *PLoS Medicine:*

Kasturiratne A, Wickremasinghe AR, de Silva N, Gunawardena NK, Pathmeswaran A, et al. (2008) Estimating the global burden of snakebite: A literature analysis. PLoS Med 5(11): e218. doi:10.1371/journal.pmed.0050218

H. Janaka de Silva and colleagues estimate that globally at least 421,000 envenomings and 20,000 deaths occur each year due to snakebite.

In this issue of *PLoS Medicine*, H. Janaka de Silva and colleagues report on a new estimate of the worldwide morbidity and mortality of snakebite, using a more thorough and rigorous search for data [6]. The researchers obtained primary data in three ways: (1) searching for publications on snakebite, (2) extraction of country-specific mortality data from databases maintained by United Nations organizations, and (3) identification of grey literature by discussion with key informants. Their new study confirms that morbidity and mortality due to snakebite are very high. The annual number of snakebites could be as high as 5.5 million, and deaths could range from 20,000 to 94,000. These estimates have a wide interval because of the limitations of the sources used and uncertainties about the primary data.

## Limitations in Epidemiological Research on Snakebite

Reporting of snakebite—and particularly envenoming—by health authorities is generally very poor in most developing countries. To evaluate snakebite incidence and mortality, researchers therefore rely upon systematic reviews of the medical literature. Most of the current, accessible primary studies use the basic method of retrospective compilation of hospital registers or statistics from medical services. Primary data may also be obtained from prospective surveys, which can give better information on symptoms, complications, or effectiveness of treatment, but such surveys take longer and are more expensive.

However, both types of health centre surveys—retrospective and prospective—only account for a proportion of all snakebites, since some patients fail to attend health centres. And in developing countries, most patients (60%–80%) who do arrive at health centres with snakebite do so after a considerable delay (sometimes several days after the bite) because they first attend a traditional healer. Delay in attending health centres has been well documented in Africa [3,7,8], and to a lesser extent in Asia [9,10] and Latin America [11,12].

One may assume that some snakebite victims die before reaching the health centre in due time (leading to underestimation of snakebite mortality), and others do not go to the health centre because they were cured (leading to underestimation of morbidity). Nevertheless, complications of snakebite leading to serious sequelae (amputations or neurologic deficits) are common. Certainly, snakebite morbidity is more likely to be underestimated than mortality because death is a less frequent outcome and probably better reported than envenoming.

An alternative study methodology uses household surveys to question a representative part of the population to estimate the incidence and mortality of snakebite in the community. This technique, recently validated by prospective follow-up of populations that confirmed its reliability [13], is a good complement to hospital surveys. However, although household snakebite surveys can be valuable and informative in helping to plan the community's need for antivenom, this method is not yet well developed.

In the new study by de Silva and colleagues, the data were obtained from a limited number of studies, which were local and scattered. The main limitation of their study, as for similar types of evaluation, is the concern about how representative it is of the actual epidemiological situation. In my own 1998 study [5], I estimated that these local surveys were fairly representative, but de Silva and colleagues believe that they are not. They argue that my assumption was undoubtedly too optimistic. Let's hope that de Silva and colleagues' study will encourage clinicians and health authorities to report snakebite cases and deaths more accurately.

## Making Antivenom More Accessible

A better knowledge of morbidity and mortality due to snakebite would lead to improved management, and it may reduce the case fatality rate and mortality (though perhaps not the incidence). Armed with better information on the global burden of snakebite, antivenom manufacturers would be able to better regulate production, and medical authorities could distribute antivenoms to where they are most useful and needed. In order to obtain better knowledge on snakebite morbidity and mortality, we need to standardize methods for collecting data, including well-designed hospital and household surveys. De Silva and colleagues' study is a preliminary, but essential, step in improving accessibility of antivenoms and the treatment of snakebite.
